# Supplementary material for: Nucleotide excision repair deficiency is a targetable therapeutic vulnerability in clear cell renal cell carcinoma
Source: Sci Rep. 2023 Nov 23;13:20567. doi: 10.1038/s41598-023-47946-4 (PMC10667362; doi:10.1038/s41598-023-47946-4)
Supplement: Supplementary file 1 — Supplementary Information. [file 41598_2023_47946_MOESM1_ESM.pdf]

Nucleotide excision repair deficiency is a targetable therapeutic vulnerability in clear cell renal cell carcinoma.

Supplementary information

Aurel Prosz[1†], Haohui Duan[2,3†], Viktoria Tisza[4,5†], Pranshu Sahgal[4,6,7,8], Sabine Topka[9,10,11], Gregory T.Klus[4,12], Judit Börcsök[1,13], Zsofia Sztupinszki[1,4], Timothy Hanlon[14], Miklos Diossy[1,4.], Laura Vizkeleti[15], Dag Rune Stormoen[16], Istvan Csabai[17], Helle Pappot[16], Joseph Vijai[9,11,18,19], Kenneth Offit[9,10,11,18,19], Thomas Ried[12], Nilay Sethi[6,7,8], Kent W. Mouw[13,20,21], Sandor Spisak[5]*, Shailja Pathania[2,3]*, Zoltan Szallasi[1,4,15]*

Affiliations

[1]Danish Cancer Institute, Copenhagen, Denmark
[2]Center for Personalized Cancer Therapy, University of Massachusetts, Boston, MA
[3]Department of Biology, University of Massachusetts, Boston, MA
[4]Computational Health Informatics Program, Boston Children's Hospital, Boston, MA
[5]Institute of Enzymology, Research Centre for Natural Sciences, Budapest, Hungary
[6]Department of Medical Oncology, Dana-Farber Cancer Institute, Boston, MA, USA;
[7]Department of Medicine, Brigham and Women's Hospital and Harvard Medical School, Boston, MA, USA;
[8]Broad Institute of Massachusetts Institute of Technology (MIT) and Harvard University, Cambridge, MA, USA
[9]Department of Medicine, Memorial Sloan Kettering Cancer Center, New York, New York.
[10]Cancer Biology and Genetics Program, Memorial Sloan Kettering Cancer Center, New York, New York.
[11]Niehaus Center for Inherited Cancer Genomics, Sloan Kettering Institute, Memorial Sloan Kettering Cancer Center, New York, New York.
[12]Genetics Branch, Center for Cancer Research, National Cancer Institute, Bethesda, MD, USA.
[13]Biotech Research & Innovation Centre, University of Copenhagen, Copenhagen, Denmark
[14]Department of Radiation Oncology, Dana-Farber Cancer Institute, Boston, MA
[15]Department of Bioinformatics, Semmelweis University, Budapest, Hungary
[16]Department of Oncology, Rigshospitalet, University Hospital of Copenhagen, Denmark
[17]Department of Physics of Complex Systems, Eötvös Loránd University, Budapest, Hungary
[18]Department of Medicine, Weill Cornell Medical College, New York,New York.
[19]Clinical Genetics Service, Department of Medicine, Memorial Sloan Kettering, New York, New York.
[20]Department of Radiation Oncology, Brigham & Women's Hospital, Boston, MA
[21]Harvard Medical School, Boston, MA

[†]These authors contributed equally

*Co-corresponding authors: Z.S. (zoltan.szallasi@childrens.harvard.edu), S.P (Shailja.Pathania@umb.edu) and S.S. (spisak.sandor@ttk.hu)

Cell-line	PTGR1 expression	IC50 (nM)	6-4PP IF
A498	+++	91.4	31.6
786O	++	395	41.8
769P	+	563.5	28.7
CAKI1	-	838	NA
RXF393	+	153	78.6
SLR26	+	381	NA
ACHN	-	355.5	NA

Supplementary table 1: PTGR1 expression, Irofulven IC50 and 6-4PP IF ratio at the 7th hour for the NER-profiled cell lines.

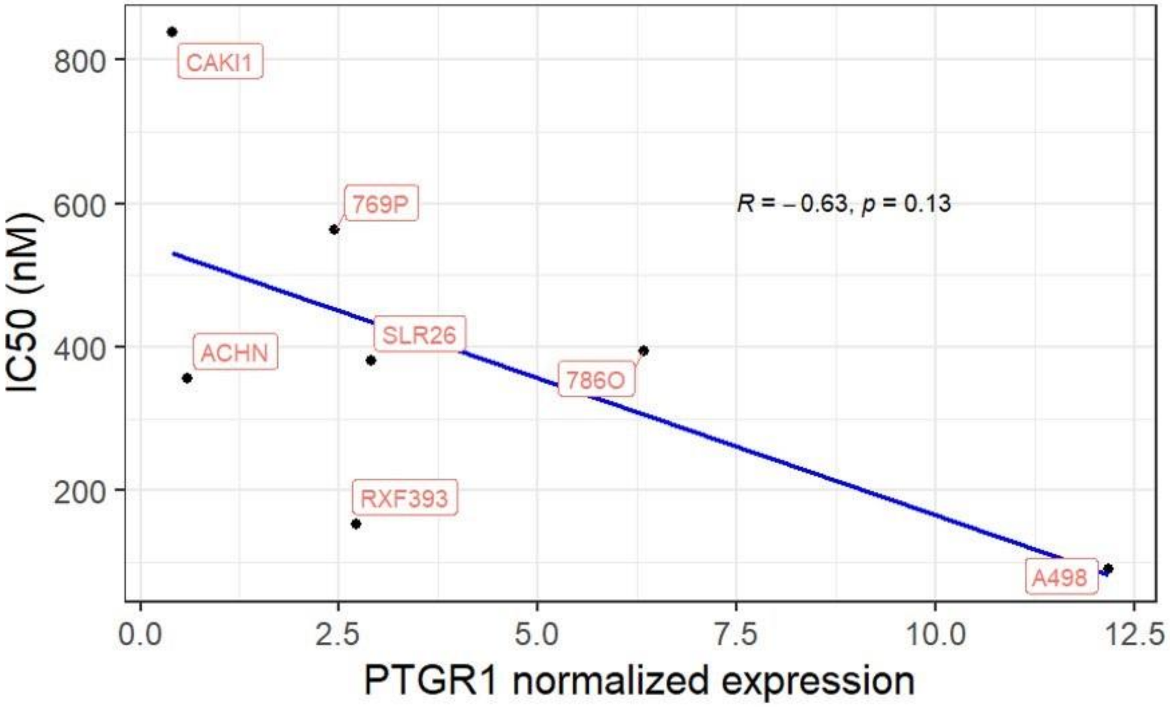

Supplementary figure 1: A negative trend can be observed between PTGR1 normalized expression and Irofulven IC50 values in renal cancer cell lines. The relationship between the

expression level of PTGR1 determined by Western Blot and the effectiveness of Irofulven in preventing the proliferation of kidney cancer cells. Elevated PTGR1 expression correlates with enhanced medication potency (lower IC50 values).

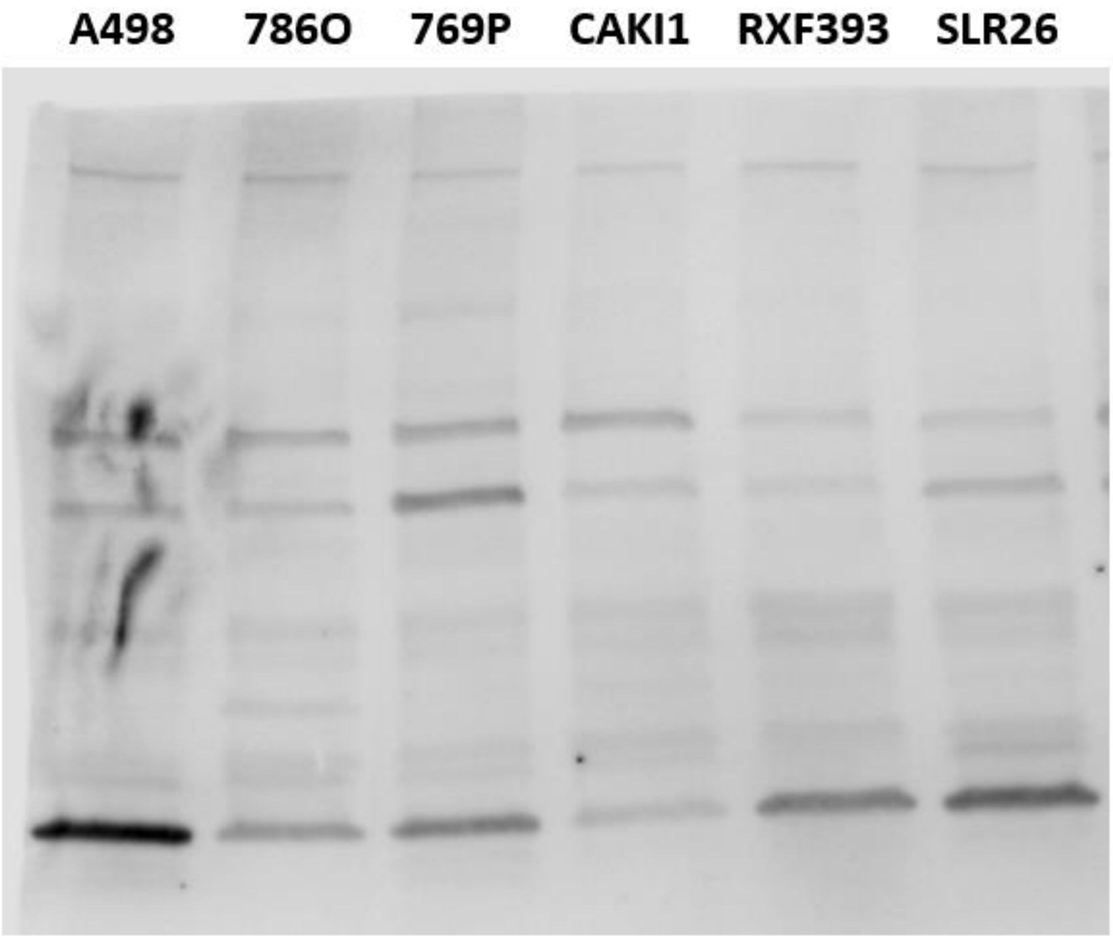

Supplementary figure 2: PTGR1 is expressed in several kidney cancer cell lines. Western blot analysis of PTGR1 expression levels in kidney cancer cell lines, where the full-length gel is shown.
